# Detection and molecular typing of epidemic *Brucella* strains among camels, sheep, and cattle in Xinjiang, China

**DOI:** 10.1371/journal.pone.0311933

**Published:** 2024-10-17

**Authors:** Liu Xingxing, Guangzhen Shi, Lidan Li, Rui Zhang, Jun Qiao

**Affiliations:** 1 School of Xinjiang Second Medical College, Karamay, Xinjiang, China; 2 College of Animal Science and Technology, Shihezi University, Shihezi, Xinjiang, China; North Carolina State University, UNITED STATES OF AMERICA

## Abstract

*Brucellosis* is a zoonotic disease that can result in symptoms including infertility, abortion, testicular inflammation, and arthritis in affected sheep and cattle. The disease can spread through a range of mechanisms, making outbreaks difficult to control such that affected farms often suffer severe economic losses. In addition, humans can be affected by brucellosis, and the number of cases continues to rise annually. As rates of brucellosis in Xinjiang, China have been increasing substantially in recent years, this study was developed to assess brucellosis seroprevalence among herds of cattle, sheep, and camels in Xinjiang through the use of common diagnostic procedures like the Rose Bengal test (RBT) and PCR. A cross-sectional approach was used to analyze the seroprevalence of brucellosis across 720 total farm animals (320 camels, 250 sheep, and 150 cattle). RBT incidence rate for brucellosis was detected in 60 serum samples, 14 (4.38%) from camels, 45 (18%) from sheep, and 1 (0.67%) from cattle. PCR was performed as a confirmatory approach for these RBT-positive samples, with 55 ultimately being confirmed to be positive 13 (4.06%), 41 (16.4%), and 1 (0.67%) samples from camels, sheep, and cattle, respectively. In this survey, sheep exhibited the highest brucellosis seroprevalence using these two analytical techniques, while cattle exhibited the lowest seroprevalence and camels exhibited an intermediate rate. AMOS-PCR analyses identified *Brucella melitensis* as the unknown bacterium in some of these samples. These results offer new insight regarding brucellosis seroprevalence among farm animals that can be used to formulate more appropriate prevention and control policies, while also improving awareness of epidemic prevention efforts, the need for routine quarantine and disinfection, the benefits of scientific breeding and management, and approaches to improving breeding efficiency for the camel, cattle, and sheep industries.

## Introduction

Reports of brucellosis in camels were first described in Russia in 1931 [1], with the most common causative strains being *B*. *abortus* and *B*. *melitensis*. Camels infected with brucellosis experience the same symptoms as with infected small and large ruminant species which are testicular inflammation, reduced milk yields, abortion, stillbirths, fetal death, and infertility that can cause significant economic losses [2]. It has also been suggested that camels show only few clinical signs [3]. Brucellosis in camels has rarely been reported in China. Brucellosis among camels in Xinjiang may contribute to the incidence of this disease in humans as they live in close proximity to their livestock and often consume camel milk in a raw form. Maintaining mixed herds of cattle, sheep, and goats is also common in some areas, increasing the odds of brucellosis cross-transmission. The complex epidemiology of this disease, insufficient information regarding the demographic characteristics of camel populations, and a lack of vaccination and other *Brucella* control programs for sheep, cattle, and goats may all play a role in the incidence of camel brucellosis. Poor control of the movement of animals across borders may also account for the prevalence of this disease in areas where ruminants and camels are reared near one another, hampering efforts to control its spread in Xinjiang. Controlling brucellosis in infected herds is vital to minimize economic losses and the risk of human zoonosis. While camels are not considered to be primary hosts for any species of *Brucella*, they are susceptible to infections with both *B*. *abortus* and *B*. *melitensis* [4], with infection spreading to husbandry and management practices, in the absence of veterinary services, limited awareness, and the free movement of pastoralists. This is consistent with results published by Teshome [5], who concluded that outbreaks of brucellosis in camels are primarily the result of mobile contact between infected animals and susceptible camel herds, and that in contact may lead to more severe epidemics of brucellosis. Similarly, Ghoneim [6] observed higher rates of brucellosis among camels in intensively farmed areas relative to free-grazing desert camels. Brucellosis caused by *B*. *melitensis* and *B*. *abortus* has been reported in all countries where camels are reared other than Australia, and the spread of disease is closely associated with breeding and husbandry practices [7].

Brucellosis is an infectious zoonotic bacterial disease that is highly prevalent throughout the world and that can be readily transmitted between animals and humans. Mammals including cattle, sheep, camels [8], pigs, and dogs are the primary source of infection. The *Brucella* genus includes the species *B*. *suis* which primarily infects pigs, *B*. *canis* which primarily infects dogs, *B*. *melitensis* which primarily infects cattle, sheep, and camels, *B*. *abortus* which primarily infects cattle, *B*. *ovis* which primarily infects sheep and rams, and *B*. *neotomae* which mainly infects desert wood mice. Three of these brucellae are susceptible to humans, namely *B*. *abortus*, *B*. *suis*, and *B*. *melitensis* [9]. While humans cannot transmit brucellosis to one another, they can develop this disease if they eat *Brucella-*contaminated foods or come into contact with animals or byproducts thereof infected with these bacteria. As diagnosing and treating brucellosis in humans is extremely difficult, it can have a substantial negative impact on affected individuals and public health systems. Despite rapid advances in medical sciences, brucellosis remains an important clinical challenge in China, with an estimated 500,000 affected patients each year throughout worldwide [10]. Ongoing improvements in the standards of living for Chinese people have continued to drive rising demand for meat products, facilitating the further spread of brucellosis. Humans most commonly develop brucellosis following contact with or ingestion of infected sheep, infected cattle, or their byproducts [11]. Brucella infections have been reported in 170 countries and regions throughout the globe [12], and brucellosis has been described across 25 provinces, cities, and autonomous regions in China, seriously adversely impacting both animal husbandry and human health [13]. Given these challenges, brucellosis has been regarded as one of the top five animal-derived zoonoses globally for over 100 years [12].

Analyses of *Brucella* species and typing can be effectively used to trace the evolution of these bacteria, providing a foundation for the epidemiological efforts necessary to control the spread of disease in cases of outbreaks among humans and livestock. *Brucella* species identification is currently performed using phenotyping and genotyping techniques. Phenotyping techniques primarily consist of biotyping, phage typing, serological typing, drug susceptibility testing, and chemical typing [14], while genotyping techniques include Multilocus Sequence Typing (MLST) [15], Restriction Fragment Length Polymorphism (RFLP) [16], Amplified Fragment-Length Polymorphism(AFLP) [17], Multiple locus variable number of tandem repeat analysis(MLVA) [18], Pulse Field Gel Electrophoresis(PFGE) [19], and Repetitive extragenic palindromic polymerase chain reaction (rep-PCR) [20]. Based on a series of factors including culture characteristics oxidative metabolism, phage lysis characteristics, antigen characteristics, and primary hosts phenotyping techniques have been used to classify *Brucella* spp. into 9 species and 19 biotypes (FAO/WHO Expert Committee on Brucellosis 6^th^ Report). The overuse of antibiotics and disinfectants has contributed to the mutation of *Brucella* sp. in response to external environmental stress, resulting in the growing emergence of atypical mutant strains that cannot be differentiated by extant phenotyping techniques, complicating efforts to prevent or control brucellosis.

In an effort to curb rising brucellosis rates, the animal husbandry and veterinary departments of the Xinjiang Uygur Autonomous Region adopted comprehensive preventative and control strategies tailored based on local conditions. These efforts are intended to prevent disease spread between animals and humans through regional joint efforts. This strategy has been effective, and there has been a downward trend in brucellosis epidemics among humans and animals [21]. However, significant increases in the frequency of the trans-regional transport of cattle and sheep have coincided with the presence of higher numbers of livestock with brucellosis such that rates of brucellosis positivity and human disease have begun to rebound [22]. These rising rates of disease have threatened livestock production and public health, increasing the challenge facing individuals seeking to prevent and control this disease [23]. There is thus a clear need to further strengthen current risk analysis, prevention, and control efforts for brucellosis among camel, cattle and sheep.

This study was developed to explore the epidemiology of brucellosis in different regions of Xinjiang to better clarify the current situation. Through the use of serological and molecular biology techniques, the spread of brucellosis among infected camels, sheep, and cattle was analyzed in the region.

## Materials and methods

### Ethical statement

As this study did not collect any new primary animal specimens, it did not require any ethical approval or written permission. Only serum samples collected through routine medical testing efforts were used herein. All clinical strains used for this study were derived from regular diagnostic tests. This work was approved by the Research and 17 Ethical Committee of Shihezi University (No. A2021219).

### Study site characteristics

Xinjiang is located in the hinterlands of the Asian-European continent at middle latitude (75°-95°E, 35°-50°N). Xinjiang has a temperate continental climate and a total area of ~1.66 million km^2^, comprising about one-sixth of the total land area of China. The geomorphology of Xinjiang consists of three mountain ranges (the Altai Mountains, the Kunlun Mountains, and the Tianshan Mountains) that stretch across the center of the region, dividing it into North Xinjiang and South Xianjing, with the Tarim Basin in the south and the Junggar Basin in the north. Many smaller valleys and basins are also situated between the mountains. The region received an average annual precipitation of 150 mm, most of which is concentrated in mountainous areas (84.3%). The Junggar Basin between the Tianshan Mountains and the Altai Mountains includes a series of low mountains collectively known as the Western Junggar Mountains in the west, while the Beita Mountains in the east extend into, forming a roughly triangular basin. The Tarim Basin, located between the Tianshan Mountains and the Kunlun Mountains is the largest basin in China with an estimated area of 530,000 km^2^.

The Xinjiang pastoral area is a major site of livestock production in China for species including Sanhe horses, Ili horses, Sanhe cattle, Inner Mongolia fine wool sheep, Xinjiang fine wool sheep, Altai big-tailed sheep, and Ningxia beach sheep. Northwestern Xinjiang has large pastures with excellent grass quality, supporting the production of milk, meat, skins, and wool products that meet the needs of locals while also being widely exported throughout China and internationally.

### Study population

For this study, samples were collected from camels (320), sheep (250), and cattle (150) in Bazhou, Shawan, Altay City, Yumin County of Tacheng, Ajetohai Ranch of Bole City, Zalmut Township of Bozhou Hot Springs, Fuhai City of Altay, and Nanhu Township of Yizhou District of Hami in Xinjiang, see Table 1.

**Table 1:** Details of animals’ species and locality.

No.	Specie	Locality	Total sample	Sample	Number of positives	Incidence rate
1	Camels	Altay	320	78	12	3.75
		Fuhai County of Altay City		82	1	0.31
		Hotan		30	0	0
		Hami		80	0	0
		Tacheng		20	0	0
		Bole		30	1	0.31
2	Sheep	Bayingolin Mongol Autonomous	250		45	18
3	Cattle	Shawan City of Tacheng district	150		1	0.67
Total			720			

### Sample collection

Samples were taken from camels, sheep and cattle from different regions of Xinjiang using random sampling method according to the proximity of the farms and the number of livestock owned, and the blood samples of camels, sheep and cattle were collected with the consent of their owners. About 5 mL of whole blood was collected from the jugular vein of each animal and labeled according to the number and location of the animals to provide representative samples from farms of different sizes. (Table 1). Samples of serum were stored at -20°C as per the recommendations of OIE [24] to prevent degradation. In total, 720 blood samples were collected from sheep, cattle, and camels in 8 regions of Xinjiang including camels from Altay, Fuhai County of Altay City, Hotan, Hami, Tacheng, and Bole, sheep from the Bayingolin Mongol Autonomous region, and cattle from Shawan City of Tacheng district. In total, 320, 250, and 150 samples were collected from camels, sheep, and cattle, respectively.

### Rose Bengal testing

Serum from blood samples collected from camels, sheep, and cattle from Bazhou, Shawan, Altay City, Yumin County of Tacheng, Ajetohai Ranch of Bole City, Zalmut Township of Bozhou Hot Springs, Fuhai City of Altay, and Nanhu Township of Yizhou District of Hami used for RBT testing as per the OIE Manual [25]. Briefly, whole blood was allowed to stand for 30 min and serum was collected, after which the antigen and serum of a brucellosis tiger-red plate agglutination test were mixed at a 1:1 ratio on a rapid test strip with appropriate positive and negative control samples. Serum was regarded as positive (+) if agglutination was visible to the naked eye within 4 min. Results were considered negative (-) if there was no agglutination and there was a uniform pink color.

### PCR

Blood genomic DNA extraction kits were used to extract DNA from collected blood samples. *Brucella* identification was then performed based on the numbers and sizes of eight products amplified via PCR. The 424 bp *Brucella* RM57 gene target gene sequence was amplified with appropriate PCR primers (P1: 5′- GCGCATTCTTCGGTTATGAA-3′ and P2: 5′- CGCAGGCGAAAACAGCTATAA-3′). Species-level molecular identification of *Brucella* isolates was performed by PCR with the following settings: 94°C for 5 min; 35 cycles of 94°C for 50 s, 60°C for 45 s, and 72°C for 35 s; 72°C for 10 min (López-Goñi I et al. 2008). Each PCR reaction included 9.3 μl of Taq PCR MasterMix (0.1 U/μl Taq Polymerase, 3 mM MgCl_2_, 20 mM Tris-HCl, 100 mM KCl, and 500 μM of each dNTP), 4 μl of template DNA, 3.8 μl of each primer, and 4.1 μl of ddH_2_O. PCR products were visualized after separation via 1.0% agarose gel electrophoresis.

### AMOS-PCR

Amplified PCR products extracted from animal samples were analyzed by AMOS (Abortus Melitensis Ovis Suis)-PCR analyses for the RM57 gene using 16 primers reported previously (Table 2). Thermocycler settings were: 95°C for 7 min; 35 cycles of 95°C for 35 s, 64°C for 45 s, and 72°C for 3 min; 72°C for 6 min. Each PCR reaction included 8 μl of primers (0.5 μl for each primer), 4 μl of template DNA, 3 μl of ddH_2_O, and 10 μl of Taq PCR Master Mix (100 mM KCl, 20 mM Tris-HCl, 500 μM of each dNTP, 0.1 U/μl Taq Polymerase, and 3 mM MgCl_2_). PCR products were visualized after separation via 1.0% agarose gel electrophoresis.

**Table 2:** Primers used for AMOS of RM57-Bruce.

Primer names	Primer sequences (5’→3’)
P1f	ATCCTATTGCCCCGATAAGG
P1r	GCTTCGCATTTTCACTGTAGC
P2f	GCGCATTCTTCGGTTATGAA
P2r	CGCAGGCGAAAACAGCTATAA
P3f	TTTACACAGGCAATCCAGCA
P3r	GCGTCCAGTTGTTGTTGATG
P4f	ACGCAGACGACCTTCGGTAT
P4r	TTTATCCATCGCCCTGTCAC
P5f	GCCGCTATTATGTGGACTGG
P5r	AATGACTTCACGGTCGTTCG
P6f	GGAACACTACGCCACCTTGT
P6r	GATGGAGCAAACGCTGAAG
P7f	CAGGCAAACCCTCAGAAGC
P7r	GATGTGGTAACGCACACCAA
P8f	CGCAGACAGTGACCATCAAA
P8r	GTATTCAGCCCCCGTTACCT

An AMOS-PCR approach was used to identify the unknown *Brucella* strain present in camel serum samples, increase in positive Brucella strains in cattle (A19), and sheep (M5). These procedures were performed with reference to the AMOS-PCR methods detailed in “Diagnostic Techniques for Brucellosis in Animals" (GB/T 18646–2018).

### Sequence comparisons

Sequence comparisons of endemic strains of *Brucella abortus* in camels with endemic strains of *Brucella abortus* in sheep and cattle were made in an effort to identify unique mutation sites.

## Results

### Serological testing

Brucellosis seroprevalence among camels, sheep, and cattle in selected regions was assessed through a cross-sectional analysis of 720 animals (320 camels, 250 sheep, and 150 cattle). RBT results led to the identification of 60 serum samples that were positive for brucellosis, including 14 (4.38%) from camels, 45 (18%) from sheep, and 1 (0.67%) from cattle. The percentages of positive detected samples are presented in Table 3.

**Table 3:** Distribution of seroprevalence of brucellosis in camels, sheep, and cattle.

No.	Specie	Total sample	Positive	Percentage(%)
1	Camel	320	14	4.38
2	Sheep	250	45	18
3	Cattle	150	1	0.67
Total		720	60	

### PCR analyses

**Fig 1:**
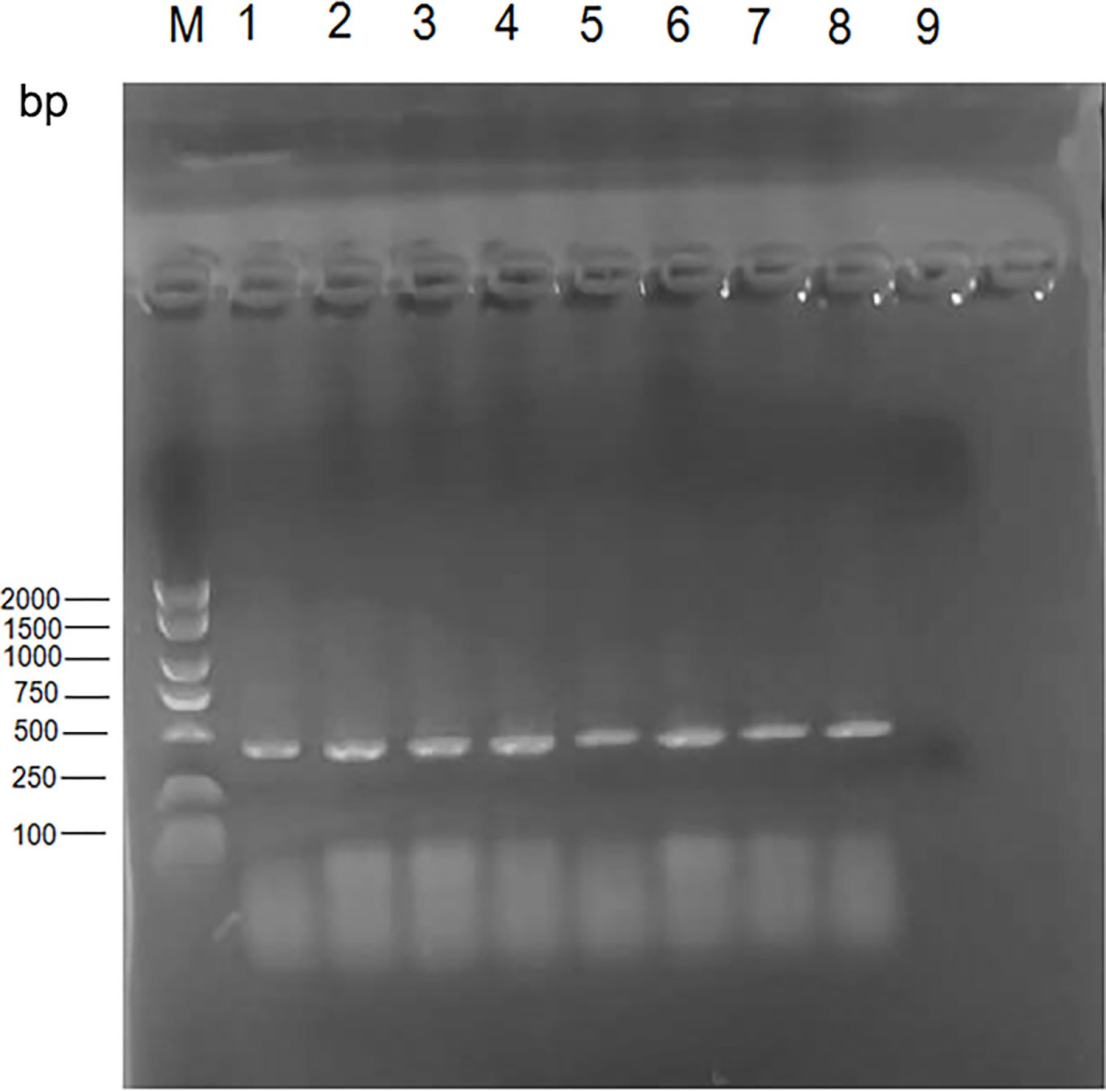
Agarose gel electrophoresis (1%) of PCR products from DNA samples. Lane M indicated DNA marker (2000bp DNA ladder); Lane 1 shows camels (Altay); Lane 2 shows camels (Fuhai County of Altay City); Lane 3 shows camels (Hotan); Lane 4 shows camels (Hami); Lane 5 shows camels (Tacheng); Lane 6 shows camels (Bole); lane 7 shows sheep(Bayingolin Mongol Autonomous); Lane 8 shows cattle (Shawan City of Tacheng district); Lane 9: Negative blank control.

DNA from camels, sheep, and cattle from different regions were amplified by PCR, yielding a 424 bp target amplicon that was then sequenced and identified through BLAST searches as corresponding to the 424 bp sequence from the *Brucella* RM57 gene (Fig 1). Confirmatory PCR testing was performed on RBT-positive samples, yielding 55 samples that were confirmed positive, including 13 (4.06%), 41 (16.4%), and 1 (0.67%) from camels, sheep, and cattle, respectively (Table 4).

**Table 4:** Detection of PCR of brucellosis in camels, sheep, and cattle.

No.	Specie	Total sample	Positive	Percentage (%)
1	Camels	320	13	4.06
2	Sheep	250	41	16.4
3	Cattle	150	1	0.67
Total		720	55	

### Identification of unknown Brucella species by the AMOS-PCR method

**Fig 2:**
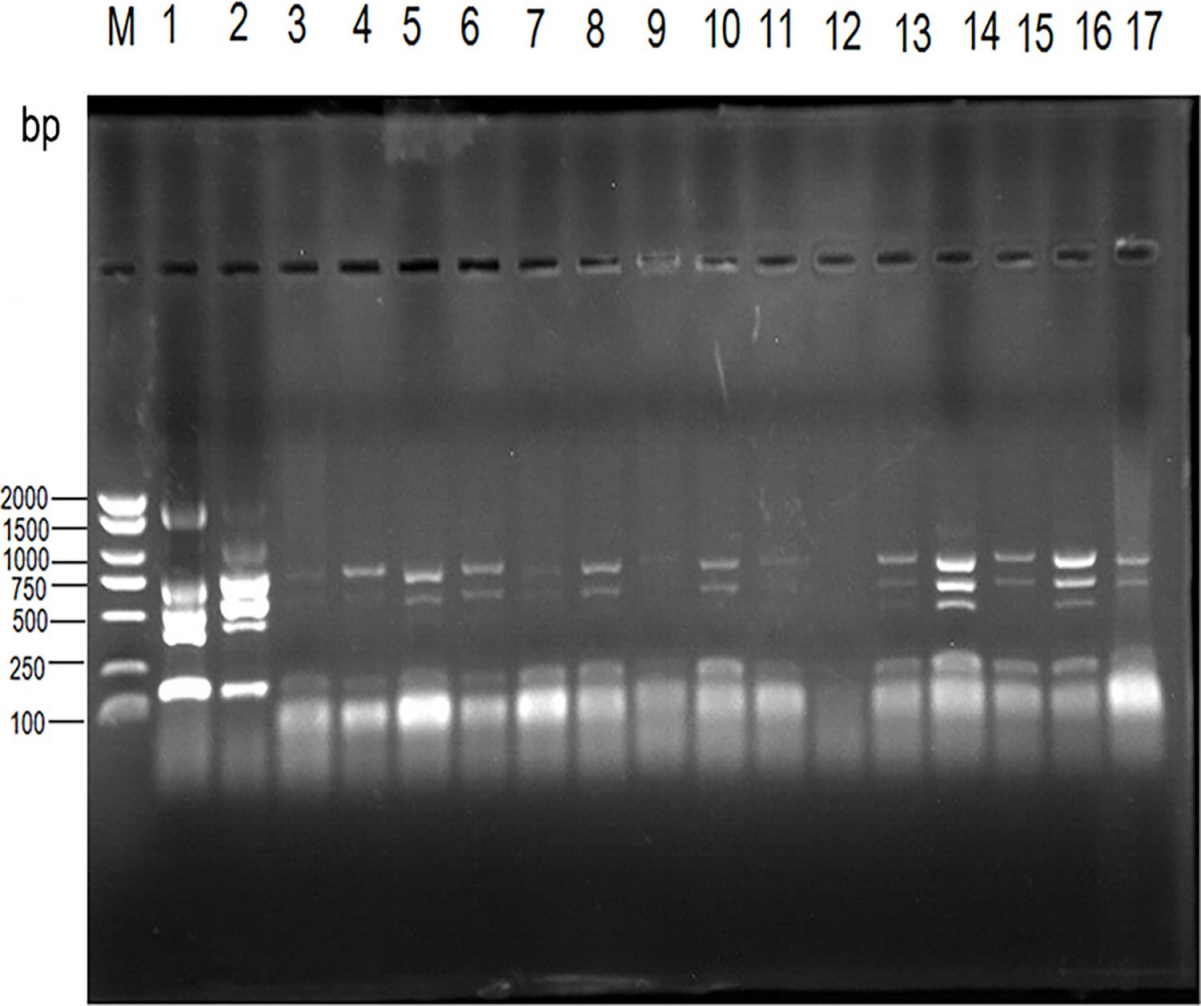
Agarose gel electrophoresis (1%) of AMOS-PCR products from DNA samples. Lane M indicated DNA marker (2000bp DNA ladder); Lane 1 shows positive control of cattle; Lane 2 shows positive control of sheep; Lane 3 shows camels (Altay); Lane 4 shows camels (Fuhai County of Altay City); Lane 5 shows camels (Hotan); Lane 6 shows camels (Hami); Lane 7 shows camels (Tacheng); Lane 8 shows camels (Bole); Lane 9 shows sheep (Bayingolin Mongol Autonomous); Lane 10 shows cattle (Shawan City of Tacheng district); Lane 11: Negative blank control.

PCR-amplified DNA from the blood of camels, sheep, and cattle was obtained via RM57-based PCR using an AMOS (Abortus Melitensis Ovis Suis)-PCR approach using 16 primer pairs documented previously. *Brucella abortus* was amplified in all nine regions with positive samples from cattle and sheep (Fig 2).

**Fig 3:**
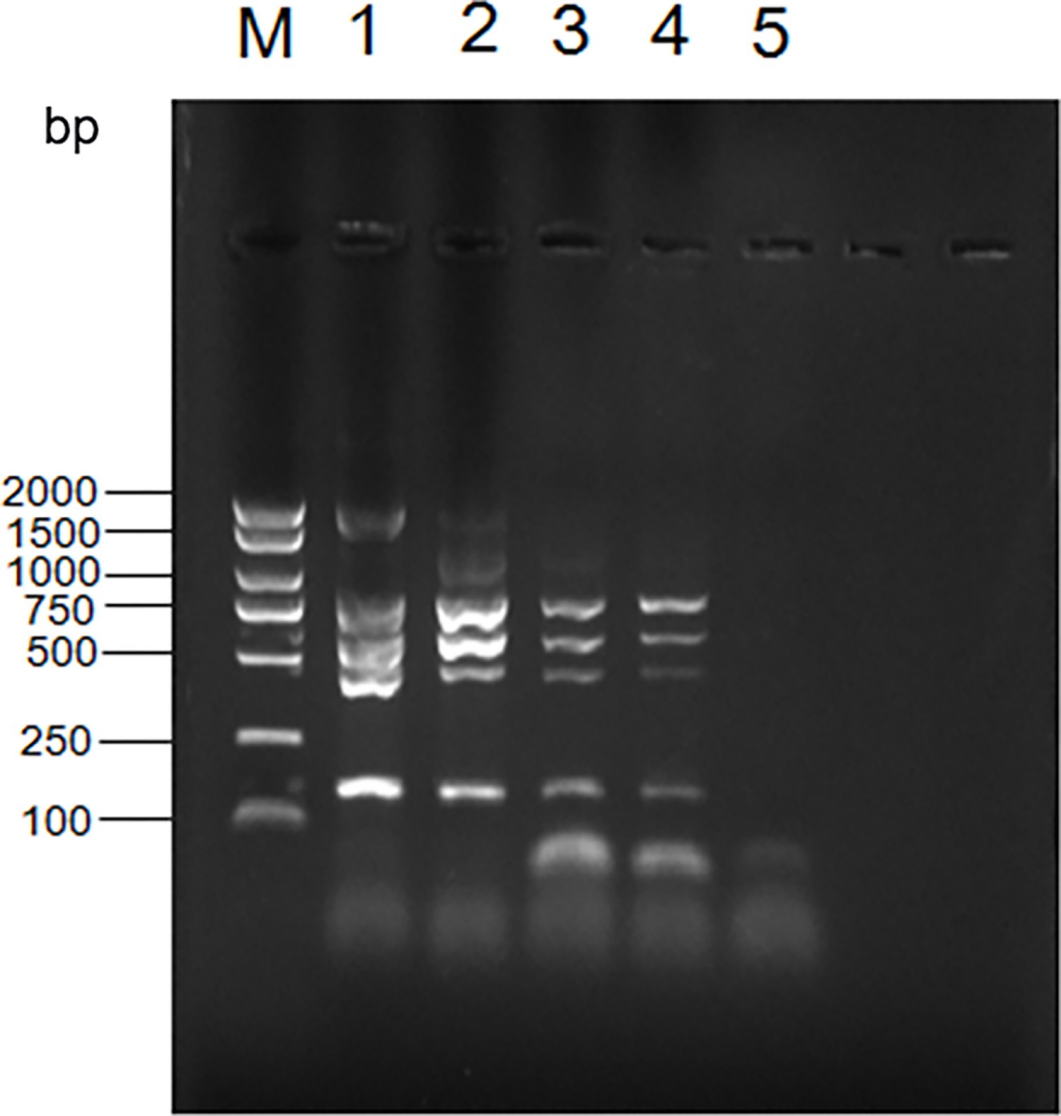
Agarose gel electrophoresis (1%) of AMOS-PCR products from DNA samples. Lane M: Indicated DNA marker (2000bp DNA ladder); Lane 1: A19(Brucella bovis); Lane 2: M5 (Brucella melitensis); Lane 3: Shows camels(unknown); Lane 4: Negative blank control.

The "Diagnostic Techniques for Brucellosis in Animals" procedure (GB/T 18646–2018), which is the national standard approach to evaluating brucellosis samples, led to the AMOS-PCR-based identification of the associated unknown bacterium as *Brucella melitensis* based on the presence of six electrophoresis bands (152 bp, 450 bp, 587 bp, 774 bp, 1071 bp, and 1682 bp). These bands same bands were identified for M5 samples, which were identified as *B*. *melitensis* (Fig 3).

**Fig 4:**
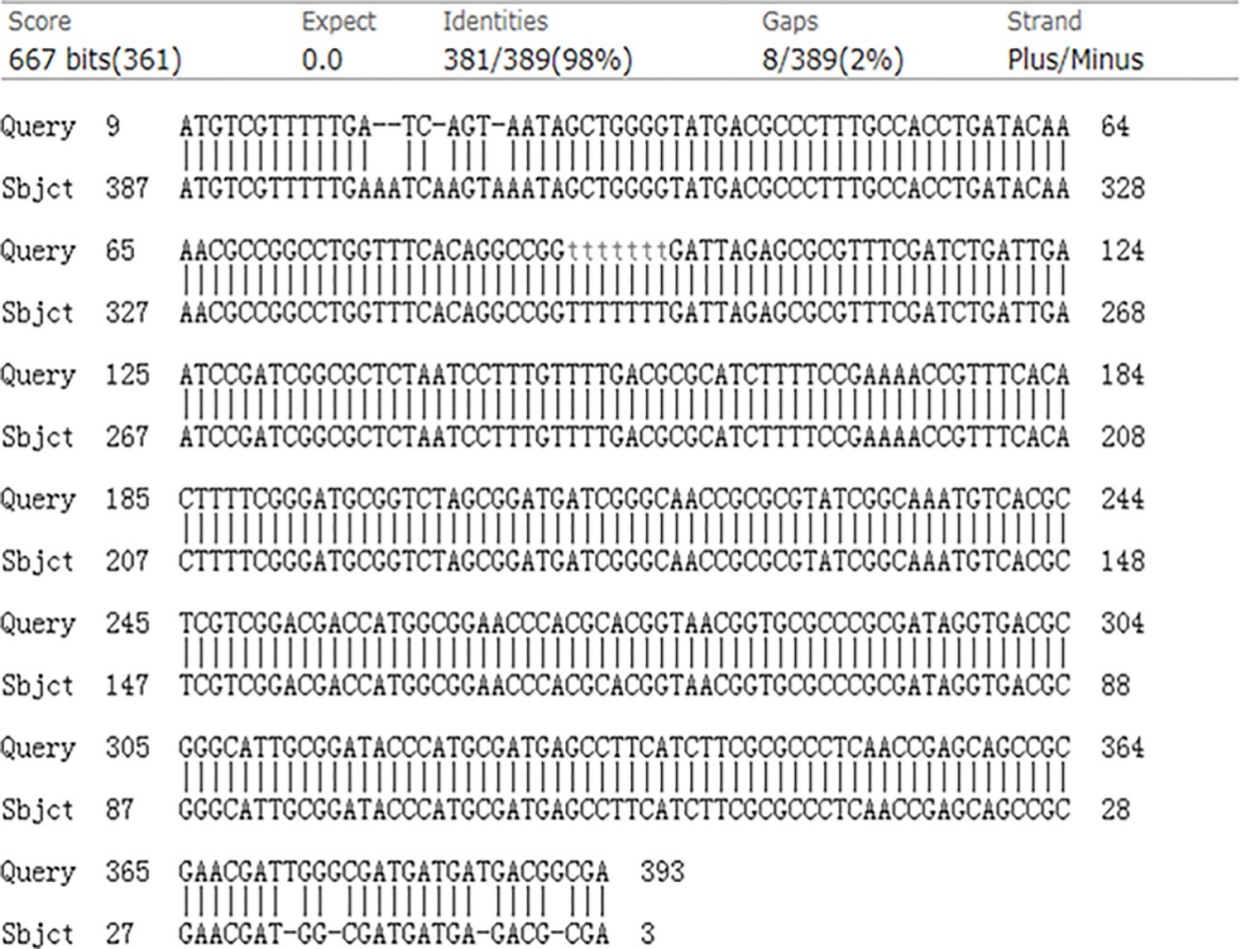
Sequence comparison of prevalent strains of Brucella abortus in camels with prevalent strains of Brucella abortus in sheep.

Sequence comparisons were performed for endemic *B*. *abortus* strains in camels, sheep, and cattle to identify unique mutations. Comparison of endemic strains of sequence comparisons of *Brucella spp*. in camels and *Brucella suis* exhibited variable sites, and no homology was observed when comparing *Brucella spp*. in camels and *Brucella bovis* (Fig 4).

## Discussion

Brucellosis is a zoonotic disease that is most common in pastoral areas such as Inner Mongolia, Xinjiang and Tibet, and is on the rise [26]. It is widely recognized that cattle are predominantly infected with B. abortus, while sheep are most frequently infected with B. melitensis [27,28]. B. melitensis and B. abortus have been mostly associated with humans, and therefore cattle and sheep are associated with human infections [29]. On the other hand, camels are susceptible to both B. melitensis and B. abortus infections although they are not the primary host of Brucella [30]. The diagnosis of brucellosis in camels is somewhat difficult and challenging as camels exhibit only a few clinical signs compared to cattle [3]. However, there is a high chance of cross-transmission between camels, cattle and sheep [31]. In Xinjiang, farmed camels are mainly taken camel milk as a drink, camel milk is an important part of people’s diet and is particularly suitable for people in arid and semi-arid regions of the world. Compared to cow’s milk, camel milk has a unique composition, with fats that are rich in medium-chain fatty acids, low in lactose, and higher concentrations of whey proteins and vitamin C [32]. Due to the habitual consumption of camel milk by the people, camel brucellosis may lead to human to develop brucellosis. Therefore, the present study was carried out to control brucellosis in infected herds using camel as the main study subject to avoid economic losses and risk of zoonotic diseases.

This study was designed to assess brucellosis seroprevalence among camels, sheep, and cattle in Xinjiang using the RBT and PCR via a cross-sectional approach. In 720 analyzed animals, RBT results identified 60 serum samples positive for brucellosis including 1 (0.67%) from cattle,14 (4.38%) from camels, and 45 (18%) from sheep, Consistent with previously published reports by Almuzaini [3] and Al-Marzooqi [33] on the increasing trend of brucellosis. PCR subsequently confirmed these results, with 55 serum samples exhibiting confirmed positivity including 1 (0.67%),13 (4.06%), and 41 (16.4%) from cattle, camels, and sheep, respectively. The greatest brucellosis seroprevalence was detected among sheep, whereas the lowest prevalence was observed among cattle, while camels exhibited an intermediate rate. AMOS-PCR detection identified the unknown bacterium in some of these samples as *B*. *melitensis*.

Efforts to control brucellosis are largely dependent on accurately determining the true prevalence of this disease through sensitive animal detection efforts and *Brucella* species identification [30]. Effective surveillance systems and control measures are vital to monitor true disease prevalence in a timely manner. Effective diagnosis thus remains the cornerstone of all control and eradication program. Diagnosing brucellosis, however, necessitates laboratory screening and confirmation using common bacteriological, molecular, and serological techniques [34,35]. Accordingly, RBT and PCR techniques were herein used to assess the prevalence of brucellosis among camels, sheep, and cattle in the Xinjiang region. Bacteriological and immunological testing are the primary diagnostic techniques for brucellosis in sheep. The RBT is a traditional screening test in which serum antibodies agglutinate with stained whole-cell preparations of dead Brucella, while PCR is often used as a form of confirmatory testing.

Brucellosis is a major zoonotic disease that is the target of prevention and control efforts in China that is prevalent among sheep flocks and causes adverse economic impacts owing to associated reductions in fertility, meat production, and milk production, while also directly threatening human health [36,37]. A major cause of brucellosis in humans is exposure to infected livestock or contaminated products derived therefrom, highlighting the importance of raising awareness as a means of preventing this disease. Brucellosis rates in Xinjiang are rising such that they represent an important public health problem. In addition to contacting infected sheep or their byproducts, humans can also develop brucellosis through slaughtering, raw mutton processing, sales, and live animal transfer [38,39], posing a serious threat to the health and economic independence of herders. The “Brucellosis Diagnosis and Treatment Expert Consensus” and “Brucellosis Diagnosis and Treatment Guidelines (for trial implementation)” established in China have provided detailed guidance for diagnosing brucellosis. The diagnostic process necessitates the integration of a range of factors including detailed questions regarding clinical manifestations and epidemiological history, together with relevant auxiliary examinations. Efforts to develop imaging technologies have led to great improvements in the rate of diagnosing brucellosis. Ultrasound analyses of the lymph nodes, abdomen, heart, and other body parts can also aid in the detection of multi-organ damage in individuals suffering from this disease. It is thus important to further study the biological characteristics of *Brucella* strains among sheep, cattle, and humans in order to clarify the dominant serotypes and pathogenetic characteristics of strains endemic as a means of better preventing and controlling this disease.

To improve efforts to prevent and control brucellosis among livestock, ensure high-quality animal husbandry, and safeguard human health, daily inspection systems have been established to enable the timely inspection, quarantine, sampling, testing, and reporting on the transfer of livestock with the potential to transmit brucellosis, particularly in cases where high rates of abortions or other suspicious factors are evident. The development and implementation of mandatory brucellosis immunization plans for cattle and sheep farms are also important, together with stronger record management and analyses to ensure the adequate density and efficacy of these vaccination efforts, providing a sound foundation for the prevention and control of brucellosis. Regular large-scale cleaning and disinfection efforts are also needed in major areas involved in the breeding, transportation, slaughter, or care of livestock in order to eliminate potential sources of infection. Work focused on breeding farms, dairy farms, and large-scale cattle and sheep farms has enabled the establishment of brucellosis-free farms and epidemic-free districts that are evaluated annually. A joint epidemic prevention and quarantine mechanism has been established to promote effective links between monitoring, mandatory immunization, and quarantine efforts. A record-keeping system for livestock and poultry transport vehicles and personnel has been fully implemented, further supporting quarantine control efforts and providing greater strength to the supervision of cross-regional live animal transfers to mitigate the spread of brucellosis. The further strengthening of knowledge regarding the prevention and control of brucellosis and the advocation of healthy consumer habits such as avoiding improperly cooked meat or raw milk can also help prevent disease outbreaks. Special efforts have been made to reach individuals involved in the breeding, transport, slaughtering, and processing of livestock in order to improve their awareness of how to prevent brucellosis, guiding them through disinfection, isolation, and other protective measures.

## Conclusion

Thus, this study shows the prevalence of brucellosis in camels, sheep and cattle, brucellosis is not only a threat to human health, but also causes economic losses to the livestock industry, therefore, it is particularly important to strengthen the detection of brucellosis for disease prevention and control. Publicizing the knowledge of disease prevention and control and advocating the habit of separate living for humans and animals will also help to prevent the outbreak of the disease.

## Supporting information

S1 File (ZIP)
